# Reducing Healthcare Costs Facilitated by Surgical Auditing: A Systematic Review

**DOI:** 10.1007/s00268-015-3005-9

**Published:** 2015-02-18

**Authors:** Johannes Arthuur Govaert, Anne Charlotte Madeline van Bommel, Wouter Antonie van Dijk, Nicoline Johanneke van Leersum, Robertus Alexandre Eduard Mattheus Tollenaar, Michael Wilhemus Jacobus Maria Wouters

**Affiliations:** 1 Department of Surgery, Leiden University Medical Center, K6-R, P.O. Box 9600, 2300 RC Leiden, The Netherlands; 2 Performation Healthcare Intelligence B.V, Sweelincklaan 1, 3723 JA Bilthoven, The Netherlands; 3 Department of Surgery, Netherlands Cancer Institute-Antoni van Leeuwenhoek Hospital, Plesmanlaan 121, 1066 CX Amsterdam, The Netherlands

## Abstract

**Background:**

Surgical auditing has been developed in order to benchmark and to facilitate quality improvement. The aim of this review is to determine if auditing combined with systematic feedback of information on process and outcomes of care results in lower costs of surgical care.

**Method:**

A systematic search of published literature before 21-08-2013 was conducted in Pubmed, Embase, Web of Science, and Cochrane Library. Articles were selected if they met the inclusion criteria of describing a surgical audit with cost-evaluation.

**Results:**

The systematic search resulted in 3608 papers. Six studies were identified as relevant, all showing a positive effect of surgical auditing on quality of healthcare and therefore cost savings was reported. Cost reductions ranging from $16 to $356 per patient were seen in audits evaluating general or vascular procedures. The highest potential cost reduction was described in a colorectal surgical audit (up to $1,986 per patient).

**Conclusions:**

All six identified articles in this review describe a reduction in complications and thereby a reduction in costs due to surgical auditing. Surgical auditing may be of greater value when high-risk procedures are evaluated, since prevention of adverse events in these procedures might be of greater clinical and therefore of greater financial impact.

**Implication of key findings:**

This systematic review shows that surgical auditing can function as a quality instrument and therefore as a tool to reduce costs. Since evidence is scarce so far, further studies should be performed to investigate if surgical auditing has positive effects to turn the rising healthcare costs around. In the future, incorporating (actual) cost analyses and patient-related outcome measures would increase the audits’ value and provide a complete overview of the value of healthcare.

## Introduction

By acknowledging the importance of reliable and valid quality information in healthcare, in the last decades, surgical audits have been initiated in several countries. Surgical auditing is a quality instrument that collects detailed clinical data from health care providers, which is used to improve quality of care by timely feedback to clinicians about their (case-mix adjusted) results and facilitate benchmarking between participating hospitals [1]. Moreover, surgical auditing provide useful information of patients usually not eligible for clinical trials and are therefore of great value for all day practice [2]. With Sweden as a pioneer [3], countries like the United Kingdom (Lothian and Borders large bowel cancer project) [4], the United States (National Surgical Quality Improvement Program) [5], and the Netherlands (Dutch Surgical Colorectal Audit) [6] developed and implemented nationwide surgical audits as well.

As raised by Michael E. Porter, the overall goal in health care should be maximizing value for patients. Value is defined as ‘the health outcomes achieved that matter to patients, relative to costs of achieving those outcomes’ [7, 8]. One of the six components of Porter’s Value-Based Health Care is ‘measurement of outcomes and costs for every patient’. As emphasized by Larsson et al. [3], measurement of outcomes by surgical audits perfectly facilitates this process of improving healthcare. With auditing, results are systematically measured and therefore might improve outcomes [9, 10].

Despite its important societal and economical position, the healthcare industry has been lagging behind regarding the availability of key data on process and outcomes of care, when compared to other industries where product evaluation is standardly embedded in the production process. Most often, focus is on patient care and quality of care instead of costs and cost reduction. Especially in the era of rapidly increasing healthcare costs, evaluation of treatments and its costs might be highly prioritized in order to reduce costs and provide good value healthcare.

In the literature so far, many articles have been published describing a relationship between surgical auditing and quality improvement. However, surgical auditing in combination with cost evaluation is described less often. Therefore, this systematic review aims to evaluate the effects of surgical auditing on hospital costs.

## Methods

### Search strategy

A systematic search of published literature before 21-08-2013 was conducted in Pubmed, Embase, Web of Science, and Cochrane Library. A specialized librarian of our institution constructed the search. No MeSH terms for studies related to ‘audit’, as in quality instrument that collects detailed clinical data from health care providers and ‘costs’, as in financial expenditure were available. Therefore, the search strategy included a variety of search terms describing ‘audit’ and ‘costs’ in order to prevent exclusion of relevant articles. Search terms on auditing (e.g., ‘audit’, ‘outcome and process assessment’, ‘NSQIP’, ‘benchmark’, ‘outcome registry’) and search terms on costs (e.g., ‘finance’, ‘economic’, ‘costs’) were combined with search terms on surgery (e.g., ‘surgery’, ‘surgeon’), see “Appendix” for the complete search.

Meeting abstracts, duplicates, and non-English-language studies were excluded.

### Selection of studies

Three authors (J. G., A. v. B., M. W.) defined the inclusion criteria. Articles were included if they met the following criteria: (1) at least one process or outcome indicator was measured or an audit was described which had been established to monitor and evaluate quality of care, (2) the indicator or audit focused on patient care within the surgical department, (3) the indicator or surgical audit itself described cost evaluation over time. Two investigators (J. G., A. v. B.) independently reviewed each title and, if applicable, the abstract was reviewed. The articles included after screening title and abstract, were evaluated by reading the complete manuscript. Disagreements on selection of a study were solved by deliberation between the two investigators or by consulting a third reviewer (M. W.). For additional relevant articles, reference lists and citations of the included studies were verified.

### Calculations

All costs are stated in U.S. dollars and inflated to 2013 using the Consumer Price Index [11], unless otherwise described. In the case of foreign currency, the currency was first converted to U.S. dollars using the yearly average currency exchanges rate [12]. If applicable, the effect on costs per patient due to surgical auditing was calculated by dividing the amount of total savings by the number of patients listed in the study.

## Results

### Search results

The systematic search revealed a total of 5,505 citations. After excluding duplicates and meeting abstracts, 3,608 articles were eligible for evaluation.

Main reason for exclusion criteria was studies revealing clinical pathways or population-based studies instead of audits with regular feedback and benchmark. Furthermore, studies not showing any information on costs were excluded.

Twenty-nine articles met the inclusion criteria on title and abstract. However, 16 articles were excluded after reading the manuscript because no surgical audit was described (inclusion criterion one). Although all remaining 13 articles described an audit in a surgical setting (inclusion criterion two), another seven articles were excluded since they did not show any data on cost reduction over time (inclusion criterion three). Some of those articles theorize about cost reduction or costs effectiveness due to surgical auditing, but do not describe actual financial calculations [10, 13–15].

**Fig. 1:**
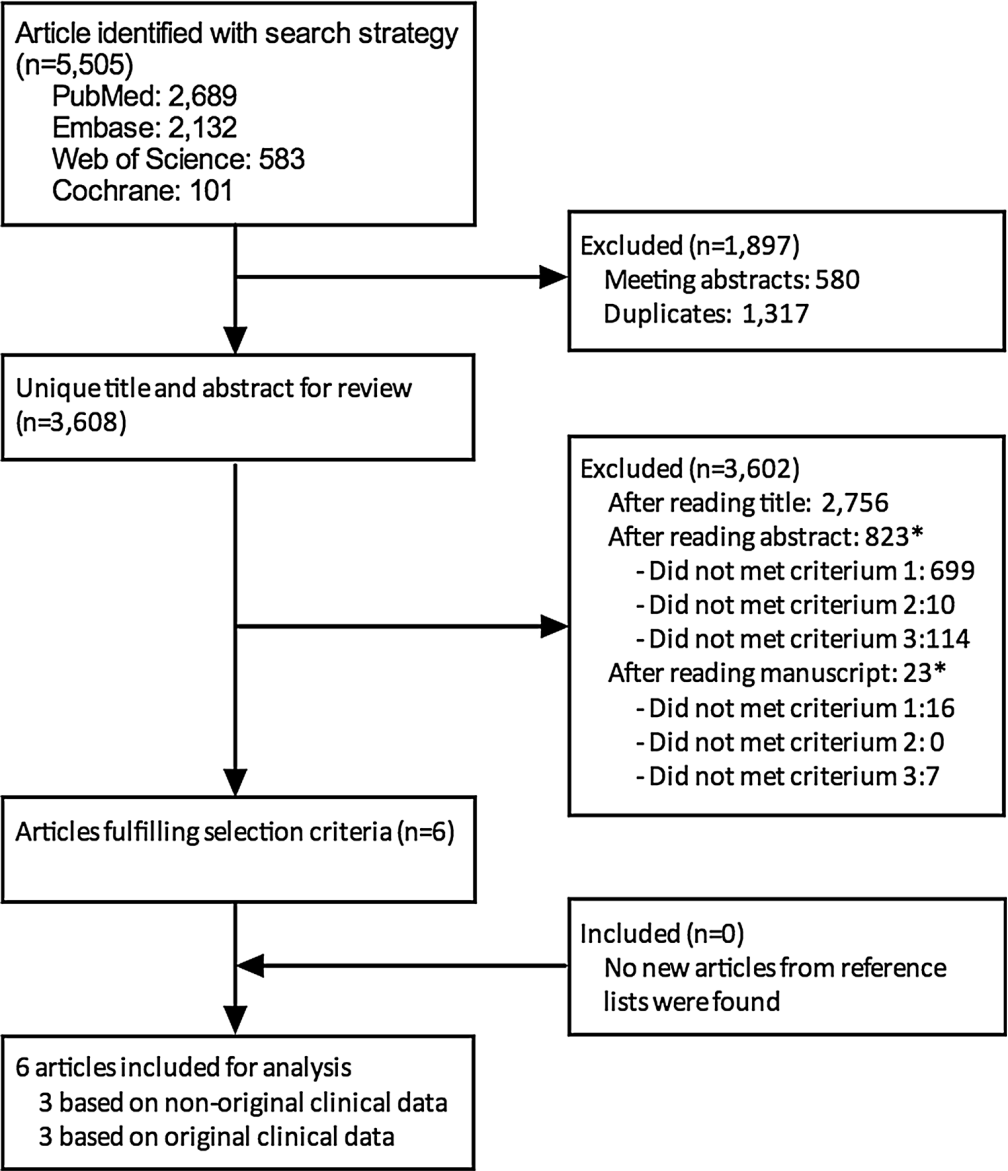
Selection process. The used strategy is outlined in “Appendix”

A total of six articles met the predefined inclusion criteria [16–21]. The reference lists of these six studies revealed no new articles. The study selection is shown in Fig. 1.

### Auditing and costs

All six studies describe a relationship between surgical auditing and cost reduction. However, three studies based their analyses on non-original clinical data [19–21] and were therefore analyzed separately (Table 1).

**Table 1:** Included articles using non-original clinical data

First author	Englesbe et al.	Gordon et al.	Larsson et al.
Year of publication	2007	2010	2013
Audit/source	Michigan Surgical Quality Consortium^a^	Literature review ‘complications colorectal cancer surgery’ and ‘effectiveness of surgical audits’	Swedish Hip Arthroplasty Register
Procedures analyzed	General and vascular surgery	Colorectal cancer surgery	Hip surgery
Setting	15 United States hospitals	Australian hospitals	Swedish hospitals
Start audit	2005	Not applicable	1979
Estimated clinical outcome^b^	3 % complication reduction based on earlier published data	50 % reduction of adverse events based on literature	Reduction of 750 hip revisions a year as compared to U.S. setting
Estimated financial outcome^b^	$936,667 (2007: $833,333) per year for 15 hospitals^c^	$24 million (2009: AU$ 30,3 million) per year for all Australian hospitals	$14.5 million (2011: $14 million) per year for all Swedish hospitals
Average patient savings	$18 (2007: $16)^c^	$1,986 (2009: AU$ 2,436)	Not described

^a^Clinical data retrieved from American College of Surgeons—National Surgical Quality Improvement Program

^b^Outcomes are estimations since these articles did not use original data

^c^Analysis based on financial data in article

#### Non-original clinical data

Englesbe et al. [20] described the potential for payers to participate in quality improvement programs by supporting 80 % of data collection costs and all of the coordinating center costs. If a reduction of surgical complications (general and vascular surgery) by 3 % per year was established by the Michigan Surgical Quality Collaborative (MSQC); the payer would save $2.81 million (2007: $2.5 million) on the program after 3 years. Gordon et al. [21] estimated the potential cost savings for colorectal surgery in Australian hospitals. Savings were attributed to a surgical self-audit system by combining existing literature on colorectal cancer surgery complications and effectiveness of surgical audit with financial data. A potential of $24.7 million (2009: AU $30.3 million) could be saved for colorectal cancer surgery in Australian hospitals each year by implementing surgical self-auditing. The third identified article by Larsson et al. [19] analyzed the potential of disease registries to improve healthcare in five different countries. Only for the Swedish Hip Arhtroplasty Register financial analyses were performed. Based on registry data (no details specified), an estimation was made that Sweden avoided 7,500 hip revisions between 2000 and 2009, if Sweden’s revision burden had been as high as that of the United States in the same period. Given costs of $19,159 (2011: $18,500) per revision (financial analyses not further specified) Larsson et al. estimated that $14,499 million (2009: $14 million) could be avoided per year in revision costs for hip surgery.

#### Original clinical data

The other three articles, based on original data, were published in the last 4 years and retrieved their clinical data from the American College of Surgeons—National Quality Improvement Program (ACS-NSQIP) (Table 2) [16–18].

**Table 2:** Included articles using original clinical data

Article characteristics			
First author	Henke et al.	Hollenbeak et al.	Guillamondegui et al.
Year of publication	2010	2011	2012
Audit characteristics			
Name	MSQC^a^	ACS-NSQIP	TSQC^a^
Procedures analyzed	Vascular surgeries	General and vascular surgeries	General and vascular surgeries
Setting	16 U.S. hospitals	1 U.S. academic hospital	10 U.S. hospitals
Start Audit	2005	July 2007	May 2008
Control period	April 2005–March 2007	Study 1: July 2007–December 2007 Study 2: July 2007–June 2008	January 2009–December 2009
Audit period	April 2007–March 2008	Study 1: July 2008–December 2008 Study 2: July 2008–June 2009	January 2010–December 2010
Summary QI program	Timely feedback of data and comparison between institutions	Not specified (start of QI program in July 2008)	Sharing surgical process and outcome data between hospitals
Clinical characteristics			
Study size			
Control	2,453 patients	Study 1: 699 patients Study 2: 1,230 patients	14,205 patients
Audit	3,409 patients	Study 1: 522 patients Study 2: 992 patients	14,901 patients
Main clinical outcomes	2 % decrease in overall morbidity	Not described	Significant reduction in: SSI, >48 ventilator hours, graft/host/flap failure, RF, wound disruption
Financial characteristics			
Financial source	Hospital accounting database	Hospital accounting database	ACS-NSQIP ROI calculator
Inclusion program costs	No	Yes (2009: $138,821 a year)	Yes (ROI calculator)
Financial analyses	Extrapolated cost savings from complication rates (earlier study)	Costs-to-charges methodology, audit group included NSQIP fee	Complication rate difference multiplied by complication costs
Average patient savings	$186 (2008: $172)	$356 (2009: $328)^b^	$238 (2009: $219)^b^

*MSQC* Michigan Surgical Quality Consortium, *ACS-NSQIP* American College of Surgeons—National Surgical Quality Improvement Program, *TSQC* Tennessee Surgical Quality Consortium, *U.S.* United States, *QI* quality improvement, *ROI* return on investment, *SSI* surgical side infection, *RF* renal failure

^a^Clinical data retrieved from ACS-NSQIP

^b^Calculation based on financial data in article

For the financial analyses, one study referred to another article [20] describing a standardized price per complication [16]. Another study used standardized complication prices based on the ACS-NSQIP return of investment (ROI) calculator [18]. The third article used ‘real costs’ using the hospital accounting database [17].

The article from Henke et al. [16] was from the same study group as Englesbe et al. [20]. Patient variables of vascular procedures (provided by ACS-NSQIP) for the original 16 hospitals of the MSQC were used. Costs of one major complication were derived from the earlier article by Englesbe et al. [20] having a fixed price of $8,287. Between the first two years and the third year, a 2 % reduction in complication rate was achieved, leading to an average cost reduction of $186 (2008: $172) per patient.

Hollenbeak et al. [17] described an improvement in cost-effectiveness with longer duration in participation in the auditing program, for both general and vascular procedures in an academic setting. Where they report a cost of $27,658 (2009: $25,471) to avoid one postoperative event 1 year after initiation of the program, these expenditures declined to 28.7 % ($7,947 (2009: $7,319)) of the initial costs 2 years after the initiation. By multiplying the savings of avoiding one postoperative event in their studied population ($9829 (2009: $9,052)) with the reduction in postoperative events (3.63 %), the program saved an average of $356 (2009: $328) per patient. Costs of the audit itself [$150,740 (2009: $138,821)], for example the NSQIP license fee and salary for a clinical nurse reviewer, were taken into account.

The study of Guillamondegui et al. [18] described improved clinical outcomes between the first and second year after implementation of a regional surgical quality collaborative of ten hospitals (the Tennessee Surgical Quality Collaborative). The reduction in complications of general and vascular procedures was translated to costs with the use of the ACS-NSQIP ROI calculator. The ROI calculator calculates costs of complications and includes costs for enrollment and participation in NSQIP. Net cost avoided between the first and second year was $2,276,911 (2011: $2,198,543) per 10,000 surgical cases. The authors outline that although the mechanisms for these changes are likely multifactorial, the Tennessee Surgical Quality Collaborative established communication, process improvement, and discussion among the members.

## Discussion

In this systematic review, a relationship between surgical auditing and reduced healthcare costs was identified. Though frequently assumed in the literature, only six articles actually described this relationship. All identified studies suggest that besides quality improvement, surgical auditing has the potential to reduce in-hospital costs.

With the continuous rise of healthcare costs, healthcare providers, insurance companies, governments, and patients demand for information and transparency on performance of hospitals. Surgical audits facilitate this process and, most important, surgical auditing might lead to improved outcomes for patients. Whether this involves orthopedic surgery [22], colorectal surgery [6], vascular surgery [16], or general surgery [9, 10, 17, 18], all show an association with improved clinical outcome. Because of the cost- and time-consuming exercise of data collection [23], the use of surgical auditing as a quality instrument will catalyze only when it proves to be cost-effective. Four of the identified articles [17, 18, 20, 21] incorporated costs of the audit itself in their calculations and therefore analyzed the actual cost-effectiveness of surgical audits. These four studies showed larger reduction in costs (due to quality improvement) compared to the audit-participation-costs, and therefore overall cost reduction was established.

Notable variation was seen in the amount of cost reduction per patient between the reported studies. Four articles using data from the ACS-NSQIP [16–18, 20] described savings in a small spectrum ranging from $18 (2007: $16) [20] to $356 (2009: $328) [17] per patient. All these studies were based on vascular or general surgical procedures, however separate financial analyses for low- or high-risk procedures were not made. Gordon et al. [21] described the highest (potential) cost reduction, reaching up to $1,986 (2009 AU$2,436) per patient. However the reported reduction of complications by 50 % accomplished with ‘self-auditing’, might overestimate clinical reality [6]. A factor that attributed to the high reduction in costs in this study might be the selection of colorectal cancer patients. In high-risk procedures, like colorectal cancer resections, the prevention of adverse events, such as anastomotic leakage, might be of greater clinical [24] and financial impact.

Remarkable, Hollenbeak et al. [17] found further cost reduction when audits were used for a longer period. This is also seen in the study by van Leersum et al. describing the first three years of the Dutch Surgical Colorectal Audit (DSCA). Between the first and the second year of the DSCA, the improvement in quality seemed to be less distinct then between the second and third year [6]. Hollenbeak et al. [17] explained the improved cost-effectiveness after a longer duration of participation by the later onset of the effect of improvement activities. Whether results further improve with even longer duration of a surgical audit should be addressed in future studies.

The exact mechanism of cost reduction by surgical auditing is sometimes hard to identify. As seen in the literature, occurrence of complications goes hand in hand with increased hospital costs [25, 26], for example due to prolonged length of hospital stay, prolonged intensive care stay and increased re-operations. The cornerstone of surgical auditing is collecting performance data and providing (benchmarked) feedback tot surgeons, leading to identification of existing problems in the care process. Knowledge of performances can facilitate quality improvement of the participating hospitals, resulting in fewer complications [6] and therefore fewer costs.

A note of caution should be made in ascribing improved quality of care (or cost reduction) to surgical auditing, since possible occurrences of secular trends not registered in the audit could influence outcomes as well [17, 27]. Also continuous development of new surgical techniques might have beneficial effect on its own. For example, the development of laparoscopic techniques may result in lower complication rates, shorter length of hospital stay, and therefore lower costs [28]. Quality improvements initiatives in individual hospitals (introduced independently from the surgical audit) might have led to overestimation of auditing results. Also participation in a registry might have some kind of Hawthorne-effect as addressed by Guillamondegui et al. [18]. Nevertheless, without availability of key data on outcomes, health care organizations are flying blind in deciding what should be targets for quality improvement initiatives or in deciding which ‘peer-hospitals’ could serve as best practice hospitals, and therefore underlying the need of audits.

An opportunity to improve insight in the value of healthcare is the introduction of more accurate cost calculations when evaluating care processes. Hollenbeak et al. [17] analyzed costs for each admission, though insights in these calculations were not given. Also, the use of the ACS NSQIP ROI calculator [18] or Diagnose-Related Groups (DRG) instrument [21] is a proxy of costs of complications, and actual costs undoubtedly vary between hospitals. Henke et al. [16], Englesbe et al. [20], and Larsson et al. [19] used a fixed price for complications by referring to earlier published articles but detailed descriptions were not given.

As suggested by Porter et al., calculating true medical costs would give a more accurate financial perspective [7], and allows one to determine the value of care. To do so, healthcare providers must measure costs at medical condition level and for the complete cycle of care. Therefore, actual costs should be used instead of declaration data. Calculating actual costs requires understanding of the resources used in patient’s care, including staff, equipment, medication, facilities, and support costs (IT, maintenance). The methodology Porter recommends to calculate the costs is time-driven activity-based costing (TDABC) [29, 30]: actual costs are calculated by identifying all clinical services in a healthcare organization and assigning both direct and indirect costs to each clinical service. Using time estimates, or actual data when available (for example in the operation room), for each service allows to specifically allocate all costs. This methodology is not commonly used in healthcare, and therefore not mentioned in literature. Articles describing an accurate translation of resource utilization into costs are scarce [31, 32]. In general, cost-based studies often use DRGs [21] or insurance claim data [33, 34] to indicate expenditures. These methods do not seem to provide an accurate economic perspective either, since DRGs do not represent ‘real costs’ but mainly depend on the classification system used [35, 36]. Moreover, since no uniformity or transparency of costs registration in various hospitals exists, most of these ‘real cost’ studies are limited to single-center settings [17, 31, 32]. If used in a multi-center setting, these costs are often retrieved from a single hospital’s accounting system and extrapolated to the other participating institutions [37].

### Limitation

No specific MeSH terms related to ‘audit’ and ‘costs’ were used. Using these two terms would result in a broad spectrum of articles describing audit as in ‘any retrospective database’ or costs as in ‘non-financial costs’. Restriction of the search (e.g., using ‘financial costs’ instead of ‘costs’) consequently increases the potential of missing relevant literature. Therefore, the search strategy included a variety of search terms describing ‘audit’ and ‘costs’ (see “Appendix”). This resulted in many articles describing clinical pathways or population-based studies without a feedback mechanism, which were excluded for this reason (exclusion criterium 1).

Because we did not find any new related articles by checking references and citations of the six included studies, the search terms seem to be adequate.

Though we used broad search terms, we only identified six articles as relevant. The three studies using original clinical data retrieved their data from 27 hospitals. Therefore, caution should be taken in generalization of our findings since results found in these hospitals may not be representative at other institutions. Finally, a major limitation of the investigated studies is the potential occurrence of publication and selection bias. Studies showing negative outcomes of surgical auditing might less likely to be submitted by the authors. Also the included studies were not designed as randomized controlled trials, therefore unattended selection bias might be introduced since the interventions (the audits themselves) were not allocated randomly to patients. The results of this systematic review should be interpreted having this limitation in mind.

### Future perspectives

We were surprised by the lack of evidence for cost evaluation of surgical auditing. As addressed by Porter, measuring clinical outcomes and costs at patient level should be embedded in the quality improvement process of healthcare [7]. In addition, the authors believe that combining clinical outcomes with patient reported outcome measures (PROMs) would provide an even stronger tool. Benchmarking hospitals on quality, costs, and PROMs could identify ‘best practices’ on all three dimensions, which will lead to higher quality of care with the use of fewer resources and less costs.

Although we only identified articles focused on hospital-related costs, registries that cover the complete patient cycle should provide better insights. Long-term complications can be identified which might cover ‘hidden’ long-term costs. For example, in colorectal cancer surgery, the creation of a defunctioning stoma shortens length of hospital stay during the initial operation and lowers short-term complications [38]. However, next to the impact on quality of life a stoma has, it also has serious long-term financial implications. Patients have a life time need for colostomy pouches and a constant risk for long-term complications [39] which are seen in up to fifty percent of the patients within ten year follow up [40]. Increasing quality and reducing costs is the fundamental base of ‘value based health care’ [7]. Therefore, covering short- as well as long-term outcomes should be aimed for all health care evaluations. While surgical auditing has become more integrated in common practice, its effectiveness on costs needs to be evaluated as well, and perhaps costs evaluation has to be incorporated in the feedback mechanisms of the audit.

## Conclusions

Ideally, the overall quality improvement related to surgical auditing should be judged with the assessment of its costs. In literature, only six articles [16–21] have been published so far, describing cost reduction due to auditing. Potential higher cost reduction is seen when the surgical audit is focused on high-risk procedures only, such as colorectal cancer surgery. Auditing could perfectly facilitate the decision-making process for reducing costs, as addressed by Porter et al. and Larsson et al. [19, 41]. Nonetheless, further studies should be performed to confirm whether surgical auditing has sustainable (long-term) effects in confining the rise in healthcare costs.

## Recommendations

In future, widespread introduction and continuous use of surgical auditing is required to evaluate and improve quality of medical care for patients. The main focus should be evaluation of high-risk procedures since prevention of adverse events in these procedures will have greater clinical and financial impact compared to low-risk procedures. Moreover, when financial outcomes are incorporated in the audit, calculating those financial outcomes should be based on actual costs, for example using time-driven activity-based costing. In the future, covering the complete cycle of care and incorporating cost analyses and patient-related outcome measures would increase the audits’ value and provide a complete overview of the value of healthcare.

Further studies describing the audit’s value should include all of the above-mentioned elements, in order to provide more robust evidence for further implementation of auditing.

## Appendix

### Pubmed, Embase, Web of Science, and Cochrane search terms

#### PubMed

Limits activated: English.

(“finance”[TIAB] OR “finances”[TIAB] OR “financial”[TIAB] OR “economics”[TIAB] OR “economic”[TIAB] OR “economics”[Majr] OR ((“costs”[TIAB] OR “cost”[TIAB]) AND (“reduction”[TIAB] OR “improvement”[TIAB] OR “health care”[TIAB] OR “healthcare”[TIAB] OR “hospital”[TIAB])) OR “euro”[TIAB] OR “dollar”[TIAB] OR “pound”[TIAB] OR “euros”[TIAB] OR “dollars”[TIAB] OR “pounds”[TIAB] OR “pound”[TIAB]OR €[tiab] OR $[tiab] OR £[tiab]).

#### AND

(“surgery”[Subheading] OR “Surgical Procedures, Operative”[Majr] OR “Surgery”[tiab] OR “Surgical”[tiab] OR “Surgeon”[tiab] OR “Surgery Department, Hospital”[Majr] OR “Specialties, Surgical”[Majr:noexp]).

#### AND

(“Outcome and Process Assessment (Health Care)”[Majr] OR “audits”[tw] OR “auditing”[tw] OR “audit”[tw] OR “Clinical Audit”[Mesh:noexp] OR “Medical Audit”[Mesh] OR “Outcome Assessment”[tiab] OR “Process Assessment”[tiab] OR “Quality Assurance, Health Care”[Majr] OR “Quality Assurance”[tiab] OR “Quality Management”[tiab] OR “Quality Assessment”[tiab] OR “benchmark”[tiab] OR “benchmarks”[tiab] OR “benchmarking”[tiab] OR “NSQIP”[text word] OR “National Surgical Quality Improvement Program”[text word] OR “outcome registry”[TIAB] OR “Outcome and Process Assessment”[TIAB] OR “Quality of Health Care”[TIAB] OR “Quality of Healthcare”[TIAB] OR “Quality Control”[Majr]).

Similar searches were performed in Embase, Web of Science and Cochrane databases.
